# Polyunsaturated Fatty Acids as Potential Treatments for COVID-19-Induced Anosmia

**DOI:** 10.3390/biomedicines12092085

**Published:** 2024-09-12

**Authors:** Yu-Han Wang, Chung-Wei Lin, Chiung-Wei Huang

**Affiliations:** 1 Department of Education, National Taiwan University Hospital, Taipei 100, Taiwan; yuhanwang0917@gmail.com; 2 Department of Education, Kaohsiung Chang Gung Memorial Hospital, Kaohsiung 833, Taiwan; 3 Department of Otolaryngology, Kaohsiung Chang Gung Memorial Hospital, Kaohsiung 833, Taiwan; 4 Department of Physiology, Kaohsiung Medical University, Kaohsiung 807, Taiwan; 5 Department of Post-Baccalaureate Medicine, Kaohsiung Medical University, Kaohsiung 807, Taiwan

**Keywords:** COVID-19-induced anosmia, polyunsaturated fatty acids, omega-3 PUFAs, omega-6 PUFAs

## Abstract

Some individuals with severe acute respiratory syndrome coronavirus 2 (SARS-CoV-2) experience anosmia, or loss of smell. Although the prevalence of anosmia has decreased with the emergence of the Omicron variant, it remains a significant concern. This review examines the potential role of polyunsaturated fatty acids (PUFAs), particularly omega-3 PUFAs, in treating COVID-19-induced anosmia by focusing on the underlying mechanisms of the condition. Omega-3 PUFAs are known for their anti-inflammatory, neuroprotective, and neurotransmission-enhancing properties, which could potentially aid in olfactory recovery. However, study findings are inconsistent. For instance, a placebo-controlled randomized clinical trial found no significant effect of omega-3 PUFA supplementation on olfactory recovery in patients with COVID-19-induced anosmia. These mixed results highlight the limitations of existing research, including small sample sizes, lack of placebo controls, short follow-up periods, and combined treatments. Therefore, more rigorous, large-scale studies are urgently needed to definitively assess the therapeutic potential of omega-3 PUFAs for olfactory dysfunction. Further research is also crucial to explore the broader role of PUFAs in managing viral infections and promoting sensory recovery.

## 1. Introduction

Coronavirus disease 2019 (COVID-19), a respiratory illness caused by severe acute respiratory syndrome coronavirus 2 (SARS-CoV-2), remains a global health concern. Despite fluctuations in its case numbers, it continues to pose a significant threat to public health. SARS-CoV-2 enters host cells by binding to the angiotensin-converting enzyme 2 (ACE2) receptor, which is expressed on the surface of various cells in the respiratory tract, including nasal goblet cells, ciliated cells, sustentacular cells, type II alveolar cells, and other epithelial cells throughout the upper respiratory tract [1,2,3]. Once inside the cell, the virus hijacks the cellular machinery, using it to replicate itself. This viral replication process can lead to cell death and the release of inflammatory factors, further triggering an immune response and contributing to respiratory illness [4,5]. The clinical presentation of COVID-19 widely varies. Some individuals may experience no symptoms at all, whereas others may develop a range of mild to severe symptoms. Common symptoms include fever, cough, fatigue, loss of taste or smell, muscle aches, headaches, and a sore throat [6,7]. In severe cases, individuals may experience shortness of breath, cyanosis, and organ failure [6,7]. Considering the persistent global health threat posed by COVID-19, there is an ongoing need for vigilant public health measures and innovative treatments to manage and ultimately overcome the pandemic.

Olfactory dysfunction has been a common symptom in COVID-19 patients, with nearly half of individuals experiencing it during earlier stages of the pandemic [8]. In some cases, anosmia persists as a long-term issue [9,10,11]. However, the prevalence of this symptom has varied with different virus variants. Notably, with the emergence of the Omicron variant, which has dominated over the last two years, the incidence of olfactory dysfunction has significantly decreased, with a global prevalence estimated at just 3.7% in adults [12]. Despite this decline, the issue remains important, as anosmia still affects a substantial number of individuals and can have lasting impacts on quality of life.

Olfaction is an inherited ability that plays a crucial role in human survival. With the rich evolution regarding the physiological behaviors and presentations of this well-developed system, the olfactory function can assist in numerous aspects of our daily lives by differentiating cozy from hazardous environments through a divergence of odors and in turn, affect the biopsychic motivation for driving subsequent movement, such as generating pleasant feelings, facilitating metabolic efficiency, and increasing libido [13,14,15,16,17]. Owing to its structural differences, our olfactory system is less functional and less sensitive to chemical stimuli than other mammals and biological organisms. Nevertheless, it still has a profound impact on human health and behavior [13,14,15,16,17,18]. The loss of smell can diminish appetite and food enjoyment and make it harder to detect potential dangers such as spoiled food or gas leaks [19,20,21]. Furthermore, it can negatively affect emotional well-being, as smell is closely associated with pleasure, memories, and social interactions [22,23,24]. All in all, the sense of smell is an important factor to consider in terms of various facets of our daily lives.

New studies have identified the probable mechanisms of olfaction. As most inhaled odorant particles are volatile and lipid-soluble, they can be easily perceived by the ingenious olfactory system through the odor receptors, which exhibit a higher affinity to lipid components [25]. Regarding the inflammatory process, it has been proven that alterations in the fatty acid composition of immune cells can drive inflammatory reactions [26]. These changes can have systemic effects, particularly when there is a variation in lipid exposure and intake [26]. During the past few decades, polyunsaturated fatty acids (PUFAs) have gained attention in inflammation mitigation [26,27]. Contrary to saturated fatty acids (SFAs) and monounsaturated fatty acids (MUFAs) that can be synthesized by the human body [28,29,30], PUFAs might even be more essential, as they must be obtained through the diet, and the downstream product of this lipid category might further aid in the alleviation of immune responses [27,31,32,33]. With the aforementioned connections, PUFAs could provide long-term benefits in disease management, potentially including the mitigation of olfactory impairment through biomolecular pathways. Despite their important role in human pathophysiology, the association between PUFAs and COVID-19-induced anosmia has yet to be fully understood. Emerging research has begun to explore the role of PUFAs in mitigating inflammation caused by COVID-19, particularly concerning anosmia [34,35]. The findings indicate that a PUFA-rich diet may help reduce the severity of olfactory dysfunction in patients with COVID-19 through various mechanisms [36,37,38,39,40]. However, it is important to note that previous studies had several limitations and shortcomings, limiting the strength of their conclusions. Notably, the 2023 randomized clinical trial by Lerner et al. found no significant effect of omega-3 PUFA supplementation on olfactory recovery in COVID-19-induced anosmia [41], underscoring the need for further studies to confirm these effects and clarify the therapeutic mechanisms involved.

This review summarizes current evidence on olfaction, anosmia, and PUFAs from both pathophysiological and biomolecular perspectives, with a focus on the association between dietary PUFA intake and COVID-19-induced anosmia. We aim to explore the therapeutic potential and underlying mechanisms of PUFAs for enhancing olfactory function in patients with post-COVID-19 anosmia. While previous research has shown some promise, recent findings include negative results that highlight the need for cautious interpretation and further rigorous studies. This research will also delve into the biomolecular pathways through which PUFAs may exert their effects and provide insights that could extend beyond olfactory dysfunction to broader strategies for managing inflammation and viral infections.

## 2. COVID-19-Induced Anosmia

### 2.1. The Physiology of Olfaction and the Etiology of Anosmia

**Figure 1:**
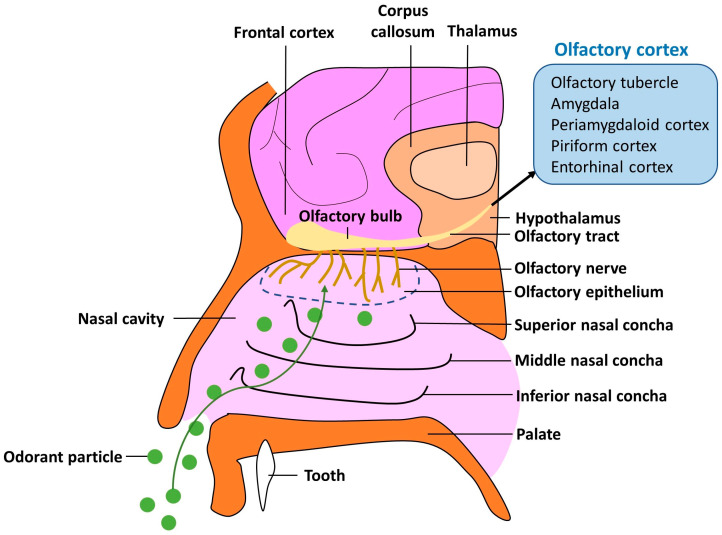
The human olfactory system. The diagram shows the intricate anatomy of the human olfactory system. Olfactory stimuli are detected, transmitted, and processed by key anatomical structures and neurobiological pathways. In the nasal cavity, the olfactory epithelium contains olfactory receptor neurons (ORNs), which detect volatile odorant molecules inhaled through the nasal passages. When an odorant binds to its receptor, it activates the receptor and initiates a series of intracellular events that generate an electric signal in the ORN. The axons of these ORNs converge to form the olfactory nerve, which transmits olfactory information from the epithelium to the olfactory bulb. The olfactory bulb consists of glomeruli formed by synapsing olfactory nerve fibers with mitral and tufted cells. Olfactory signals are processed and refined in the olfactory bulb located on the ventral surface of the brain. Subsequently, the processed signals are transmitted from the olfactory bulb to the olfactory cortex through the olfactory tract. The olfactory cortex includes several regions (shaded blue). The olfactory tubercle integrates olfactory information with motivational and emotional contexts. The amygdala emotionally responds to olfactory stimuli. The periamygdaloid cortex associates olfactory signals with perception and memory. The piriform cortex distinguishes smells. The entorhinal cortex plays a critical role in memory formation, spatial navigation, and the integration of olfactory information with other sensory inputs. Through these complex and highly coordinated pathways, the brain is able to detect, identify, and respond to a wide variety of odors, linking them to past experiences and emotions.

Olfaction is a critical physiological system. The olfactory system comprises the olfactory epithelium, olfactory nerves, olfactory bulbs, olfactory tracts, the olfactory cortex, and functionally associated subcortical structures. Olfactory tract fibers directly or indirectly terminate in the prefrontal cortex, limbic system, hypothalamus, and reticular formation of the brain stem (Figure 1) [18,42,43]. These connections generate our sense of smell and mediate behavioral responses to different odors [14,15,16,18]. Although olfaction in humans is not as sensitive or functional as that in other mammals and reptiles, it plays an essential role in hazard detection, the evocation of emotions and memories, the enjoyment of alimentary pleasures, and sexual satisfaction [13,14,15,16]. Gaining a better understanding of how olfactory information is processed and utilized for these essential functions can help mitigate potential negative consequences.

Anosmia refers to the complete loss of smell, distinct from hyposmia, which is defined as reduced ability to smell and detect odors [44,45,46]. Individuals who lose their sense of smell experience significantly diminished quality of life. Moreover, patients with olfactory impairment may struggle to discriminate among flavors, lose interest in eating, and in extreme cases, suffer from poor nutrition or depression [15,16,47]. In addition, the inability to detect warning odors, such as smoke, spoiled food, toxins, and gas leaks, can place them in dangerous situations [19,20,21]. Undoubtedly, olfaction is intimately associated with our physical and mental well-being.

### 2.2. How Does COVID-19 Cause Anosmia?

COVID-19-induced anosmia, or smell loss due to COVID-19 infection, is a common symptom of the disease. In most cases, COVID-19-induced anosmia is temporary, and smell will return within 7−14 days [48,49,50,51]. However, some people may experience long-term anosmia, which can last for months or even years [9,10,11]. The varied clinical presentations of COVID-19-induced anosmia, ranging from temporary to long-term impairment, reflect a complex interplay of pathological and physiological factors affecting olfactory function.

COVID-19 mysteriously causes anosmia. The virus is believed to damage the supporting cells [49,52] in the olfactory epithelium by entering through ACE2, the receptor for SARS-CoV-2. [10,53,54]. This damage disrupts the normal turnover of supporting cells and the function of olfactory receptor neurons, leading to anosmia. The Omicron variant, with its new mutations, likely reduces receptor binding affinity and entry efficiency to the supporting cells, which may account for its decreased impact on olfactory function [55]. Additionally, other mechanisms, such as inflammatory blockage of the olfactory cleft [10,56], can cause apoptosis of olfactory receptor neurons as a host defense mechanism [57], and elevated levels of the proinflammatory cytokine TNF-α in the olfactory epithelium [10,58] may also contribute to the development of olfactory dysfunction. Chronic inflammation, in particular, has been implicated in the persistence of smell loss long after the acute phase of COVID-19 [59,60]. Over the past few years, significant progress has been made in understanding the complex combination of pathophysiological factors that contribute to COVID-19-induced olfactory loss, highlighting the multifaceted nature of this symptom and the need for continued research.

**Figure 2:**
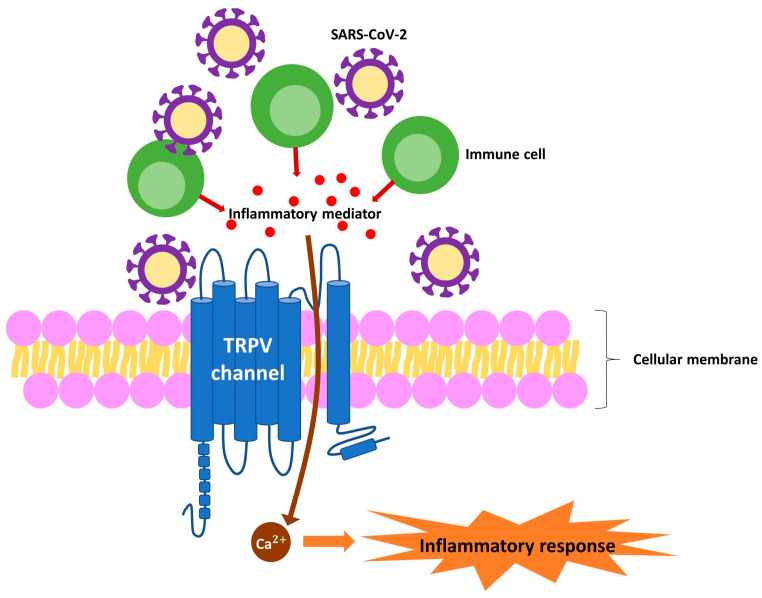
The role of transient receptor potential vanilloid (TRPV) channels in the pathogenesis of coronavirus disease 2019 (COVID-19)-induced anosmia. TRPV channels are integral to sensory perception, immune responses, and inflammation. These channels, which are found in various tissues, detect physical and chemical stimuli. TRPV channel activation leads to calcium influx, which triggers the release of inflammatory mediators. This process contributes to a cytokine storm and subsequent tissue damage. In the context of COVID-19, TRPV channels play a pivotal role in the disease pathogenesis, including the development of anosmia (loss of smell). TRPV1 activation causes neurogenic inflammation in sensory neurons. Neuropeptides released within the olfactory epithelium and bulb, such as calcitonin gene-related peptide and substance P, may damage olfactory receptor neurons, resulting in olfactory dysfunction. In addition, TRPV4 is involved in the regulation of vascular permeability and inflammatory responses. The overactivation of TRPV4 can increase inflammation and exacerbate damage to olfactory neurons and supporting cells, further contributing to anosmia. Considering their role in inflammation and immune responses, modulating the activity of TRPV channels, particularly TRPV1 and TRPV4, offers potential therapeutic strategies to mitigate anosmia and improve olfactory function in affected individuals.

Recent studies have demonstrated that the transient receptor potential vanilloid (TRPV) channel has been implicated in COVID-19-induced anosmia, with studies indicating its potential role in the pathogenesis and treatments of the condition [61,62,63]. TRPV channels are involved in various sensory processes, including pain, temperature sensation, and inflammation [64,65,66,67]. According to the studies, TRPV1 and TRPV4 were both expressed in the olfactory epithelium, with TRPV1 located near olfactory receptor neuron (ORN) axons and TRPV4 in the basal layer [61,68,69]. TRPV1 stimulated progenitor cell proliferation, whereas TRPV4 modulated their proliferation and maturation [61,69,70]. The interaction between the SARS-CoV-2 spike protein and TRPV channels may lead to dysregulation of the latter, contributing to anosmia [63,71]. However, the exact mechanisms underlying COVID-19-induced anosmia have yet to be fully understood, with inflammation and damage to the olfactory system being proposed as potential factors (Figure 2) [52,72,73,74]. Recent progress has shed light on these mechanisms, emphasizing the role of supporting cells in the olfactory epithelium [49,52]. It is important to note that the olfactory epithelium is designed to regularly replace both olfactory neurons and their supporting cells [49,52]. Understanding these processes is crucial for developing targeted therapeutic strategies that address the root causes of COVID-19-induced anosmia.

### 2.3. Current Treatments of COVID-19-Induced Anosmia

Recent studies have investigated various treatments for COVID-19-induced anosmia, exploring potential mechanisms such as inflammation of the olfactory clefts and damage to the olfactory epithelium or the central nervous system [10,49,53,54,56,57,58,73]. Understanding these mechanisms is crucial for the development of effective treatment strategies. The treatment approaches currently being investigated include olfactory training, anti-inflammatory agents, and neuroprotective agents [41,75,76,77,78,79,80,81]. Olfactory training, often combined with a short course of oral corticosteroids, is currently recommended for the management of post-COVID-19 olfactory dysfunction [81,82]. Olfactory training involves repetitive exposure to specific odors. This is believed to enhance the regenerative capacity of the olfactory system and improve neuronal targeting [83,84,85,86]. This training can stimulate olfactory neurons and promote olfactory function recovery over time.

As excessive inflammation plays an important role in the persistence of anosmia, anti-inflammatory agents are promising as potential treatments. For example, corticosteroids [11,81,87,88] are used to reduce inflammation in the olfactory clefts and may help restore the sense of smell [89,90,91,92]. A combination therapy involving doxycycline, a systemic corticosteroid, and a topical nasal steroid has been demonstrated to lead to rapid recovery in most patients compared with a control group [11,77]. Such a combination leverages the anti-inflammatory properties of these agents to reduce inflammation and promote healing in the olfactory system [11,77]. However, the effectiveness of intranasal corticosteroid sprays alone remains controversial, with some studies questioning their efficacy in treating anosmia [87,88]. Alternative treatments, such as acupuncture, have also been investigated [93]. Research suggests that acupuncture enhances olfactory function by downregulating cytokines associated with inflammation and improving nasal ventilation through sympathetic nerve dominance [93]. This approach could provide a viable option for patients who are not suited for conventional medical treatments [93]. Dietary interventions with anti-inflammatory properties, such as vitamins C, D, E, and omega-3 fatty acids, were also proposed [34,94]. A recent study has demonstrated that oral vitamin A combined with a nasal steroid spray can reduce the duration of post-COVID-19 anosmia [95]. However, it shows no significant smell score improvement compared with olfactory training alone over 4 weeks [95]. Overall, while various treatments regarding anti-inflammation show potential in treating COVID-19-induced anosmia, their effectiveness varies, and further investigation is warranted.

Considering the involvement of the neuronal pathway in COVID-19-induced anosmia, neuroprotective agents might exert beneficial effects. One promising treatment is ST266, an acellular secretome therapy that has shown potential in treating long-standing anosmia in a case study [75]. This treatment involves a biological substance containing various growth factors and cytokines, which may help repair damaged tissues and promote neural regeneration [75,96,97]. Similarly, intranasal insulin is emerging as a novel approach for the treatment of post-COVID-19 anosmia [76,98,99,100]. As insulin increases the production of growth factors, it utilizes the neuroprotective and regenerative properties of these growth factors to aid in the recovery of olfactory function [76,98,99,100,101]. Another promising approach is platelet-rich plasma therapy, which aims to regenerate damaged olfactory neurons and improve overall function [102,103]. In addition, B-complex vitamins and olfactory training have been shown to help treat COVID-19-induced anosmia in a single case study [104]. The mechanisms are still being scientifically elucidated, but the vitamin B complex acts as a potential supplementation for improving the regeneration of affected nerves [105,106,107]. Among the numerous clinical trials targeting anosmia therapy, neuroprotective and anti-inflammatory agents, such as palmitoylethanolamide and luteolin (PEA–LUT) and cerebrolysin, a mixture of porcine-derived neuropeptides and free amino acids, have shown considerable promise [78,108,109,110,111,112]. Previous studies have reported that they may promote an anti-inflammatory profile and support neural repair and regeneration [78,108,109,110,111,112]. Furthermore, the neuroregenerative potential and antioxidant properties of 13-cis-retinoic acid are being investigated for their therapeutic applications in anosmia [80,113]. 13-Cis-retinoic acid is believed to enhance neural repair and reduce oxidative stress, which can contribute to olfactory recovery [80,113]. Overall, these treatments offer a potential option for post-COVID-19 olfactory dysfunction, but further research is needed to assess their safety and efficacy.

**Figure 3:**
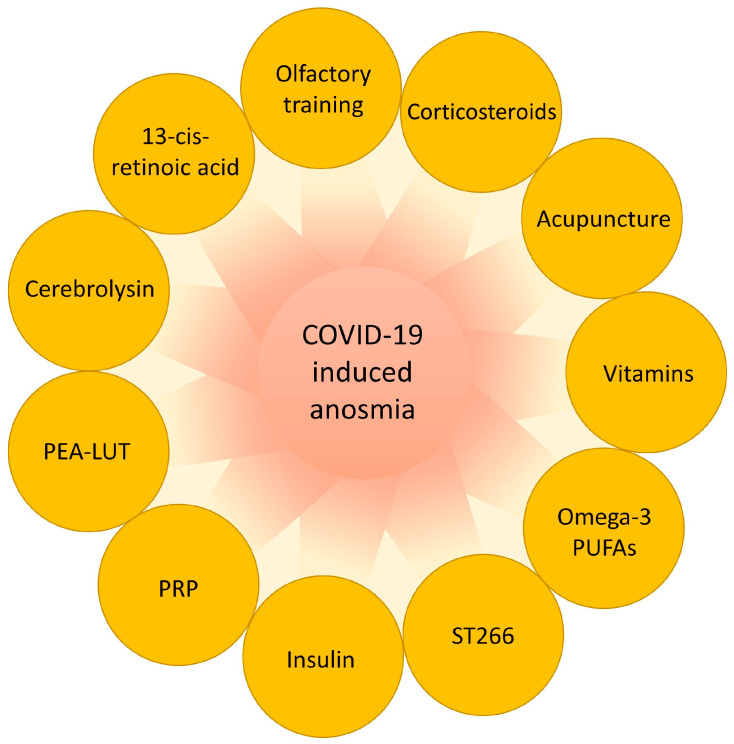
Current treatments of coronavirus disease 2019 (COVID-19)-induced anosmia. A variety of approaches are employed to treat COVID-19-induced anosmia, including olfactory training, corticosteroids, acupuncture, vitamins, omega-3 polyunsaturated fatty acid (PUFA) supplements, ST266, insulin, platelet-rich plasma (PRP), palmitoylethanolamide–luteolin (PEA–LUT), cerebrolysin, and 13-cis-retinoic acid. Olfactory training, which involves repeated exposure to different odors, helps stimulate olfactory function by re-engaging and retraining the olfactory system to recognize and distinguish various smells, thereby enhancing sensitivity and functionality. Corticosteroids reduce inflammation and nasal congestion, therefore improving olfactory function by minimizing the inflammatory response that impairs olfactory receptors. Acupuncture has shown potential in the treatment of anosmia by activating the central nervous system and influencing anti-inflammatory and immunomodulatory mechanisms. Vitamins and 13-cis-retinoic acid possess anti-inflammatory properties and influence cellular processes involved in receptor regeneration and neural plasticity, further promoting olfactory system recovery. Omega-3 PUFA supplements, known for their anti-inflammatory properties, help alleviate sensory disorders by reducing inflammation and supporting olfactory system regeneration. ST266 has shown the effective reversal of COVID-19-induced anosmia, although its mechanism of action remains unclear. Intranasal insulin may improve olfactory function by increasing the growth factor levels in the olfactory epithelium, supporting the regeneration and maintenance of olfactory neurons. PRP has shown potential in peripheral nerve regeneration by promoting vascular and axonal growth through growth factors and modulating inflammatory responses in the microenvironment. Cerebrolysin and PEA–LUT provide neuroprotection and modulate neuroinflammation, thus preserving and restoring olfactory function. Overall, these treatments exhibit anti-inflammatory, regenerative, and neuroprotective activities against COVID-19-induced anosmia.

Most patients with COVID-19-induced anosmia will fully or partially recover over time, although the duration can widely vary among individuals [114]. While these findings are encouraging, the continued development of targeted therapies remains a crucial area of investigation. The identification and validation of effective treatments are essential for improving outcomes for patients with COVID-19-induced anosmia (Figure 3). Further investigations should aim to optimize the dosage and administration methods, explore the synergistic effects of combined therapies, and conduct long-term evaluations to understand the robustness of the benefits and potential side effects.

## 3. Biomolecular Mechanisms of PUFAs

### 3.1. The Structure and Characteristics of PUFAs

A gram of fat provides nine kilocalories, more than twice the energy provided by carbohydrates or proteins, making it a crucial energy source for humans [115,116,117]. Fat is stored in adipose tissue and acts as a long-term energy reserve [118,119]. When the body needs energy, it breaks down these stored fat molecules into usable energy, offering a sustained fuel source for various bodily functions [119,120]. Additionally, fats are essential for maintaining physiological functions such as cell membrane integrity, hormone production, and thermal insulation [29,121]. In addition, fats facilitate the absorption of fat-soluble vitamins (A, D, E, and K) [122]. On the basis of their chemical structure, particularly the degree of saturation and the number of double bonds in their fatty acid chains, fats are categorized as SFAs, MUFAs, or PUFAs [123,124,125]. SFAs, which contain only single bonds, are abundant in animal fats and certain plant oils and are solid at room temperature [123,125]. MUFAs, found in olive oil, avocados, and some nuts, contain one double bond, which provides flexibility and makes them liquid at room temperature [126,127,128]. PUFAs, found in fish, flaxseeds, and walnuts, have two or more double bonds, which enhance their fluidity. These fatty acids also offer neuroprotective benefits [129,130]. Despite these structural differences, SFAs, MUFAs, and PUFAs each play crucial roles in energy provision, hormone production, supporting cell membrane integrity, the absorption of fat-soluble vitamins, and the enhancement of neuroprotection. Considering the distinct functions and benefits of each type of fat, a balanced fat intake is essential to support overall health and well-being.

According to the position of the first double bond from the methyl terminus of the carbon chain, PUFAs can be subdivided into two main classes, omega-3 and omega-6 [131]. The main omega-3 PUFAs in food sources are α-linolenic acid (ALA) (all-cis-9,12,15-octadecatrienoic acid; C_18_H_30_O_2_ and molecular weight (MW): 278.4 g/mol), eicosapentaenoic acid (EPA) (all-cis-5,8,11,14,17-eicosapentaenoic acid; C_20_H_30_O_2_ and MW: 302.5 g/mol), and docosahexaenoic acid (DHA) (all-cis-4,7,10,13,16,19-docosahexaenoic acid; C_22_H_32_O_2_ and MW: 328.5 g/mol). While ALA is abundant in linseed, rapeseed, canola, and soybean, the main sources of EPA and DHA are deep-sea fish, algae, and supplements [132,133]. Evidence suggests that omega-3 PUFAs have antioxidant and anti-inflammatory properties [134,135]. Conversely, omega-6 PUFAs in food sources include linoleic acid (LA) (all-cis-9,12-octadecadienoic acid; C_18_H_32_O_2_ and MW: 280.4 g/mol) and arachidonic acid (AA) (all-cis-5,8,11,14-eicosatetraenoic acid; C_20_H_32_O_2_ and MW: 304.5 g/mol). The common dietary sources of omega-6 PUFAs are nuts, avocados, olives, and various oils, such as sunflower, cottonseed, and corn oils [133,136]. The intake of fats containing omega-6 PUFAs, predominantly LA, provides precursors to arachidonate, which is the parent compound of eicosanoids, including prostaglandins, thromboxanes, leukotrienes, and specialized pro-resolving mediators (SPMs) [137] (Table 1). As omega-3 PUFAs are known for their anti-inflammatory properties and omega-6 PUFAs play a role in eicosanoid production, a deeper understanding of their interaction and combined effects could lead to targeted interventions for olfactory dysfunction.

### 3.2. The Biosynthetic Pathways of PUFAs

**Figure 4:**
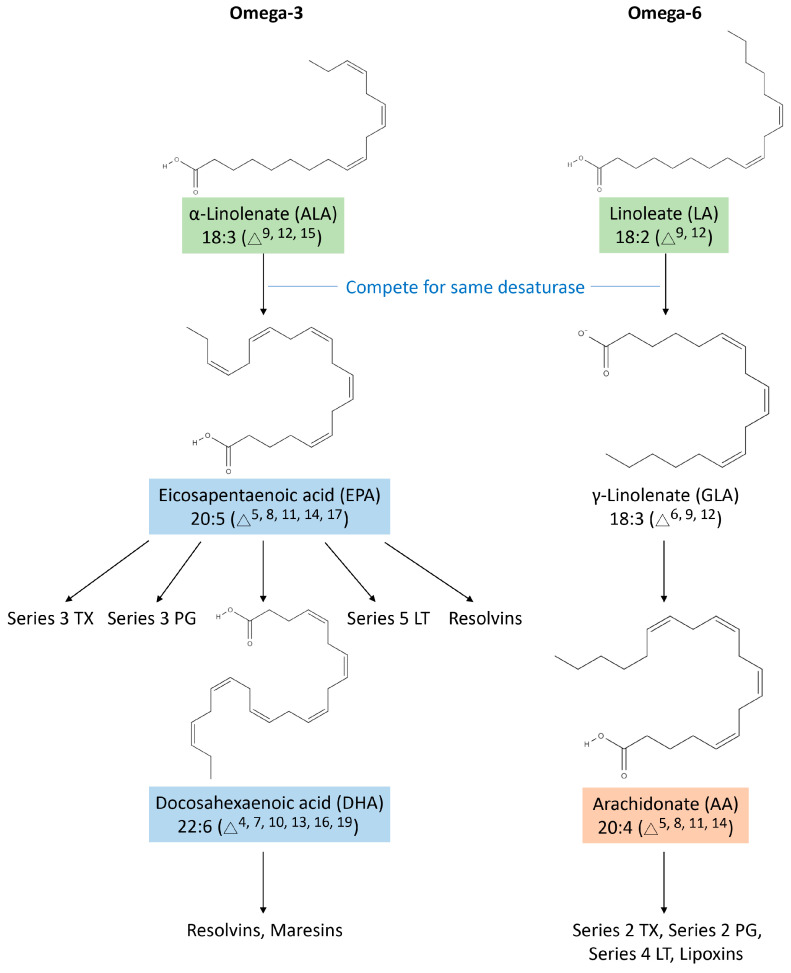
Biosynthetic pathways and interactions of omega-3 and omega-6 fatty acids and their derivatives. Omega-3 and omega-6 fatty acids interact competitively and follow intricate biosynthetic pathways. In terms of omega-3 fatty acids, alpha-linolenic acid undergoes desaturation and elongation to be converted into eicosapentaenoic acid (EPA). As a precursor for series-3 thromboxanes (TXs), series-3 prostaglandins (PGs), series-5 leukotrienes (LTs), and resolvins, EPA is vital to anti-inflammatory and pro-resolving activities. EPA can be further converted into docosahexaenoic acid, another substance that acts as a precursor for resolvins and maresins and plays a crucial role in resolving inflammation and maintaining cellular homeostasis. Contrarily, linoleic acid, an essential omega-6 fatty acid, is metabolized into γ-linolenate and finally, arachidonic acid (AA). AA plays an important role in the production of series-2 PG, series-2 TX, and series-4 LT, which contribute to proinflammatory responses and immune responses. As omega-3 and omega-6 fatty acids compete for the same desaturase enzymes, this highlights the importance of maintaining a balanced diet. These fatty acids are essential in modulating inflammation processes and promoting overall health. Excessive intake of omega-6 fatty acids relative to omega-3 fatty acids can exacerbate inflammation and associated health disorders. Therefore, understanding the interaction of these metabolic pathways is pivotal for maintaining a healthy and anti-inflammatory state.

In PUFAs, LA and ALA have the fewest carbon atoms, so they can further be elongated by enzymes and converted into other fatty acids [141,143], providing membrane fluidity and eicosanoid synthesis [144,145]. Due to the limited activity of desaturase enzymes, mammals cannot endogenously make LA or ALA, so they need to be included in their diet as essential fatty acids (EFAs) [145,146]. As a result of the action of specific enzymes, LA and ALA can be desaturated and elongated, resulting in longer carbon chains [141,143]. These elongated PUFAs can then be further converted into other fatty acids, which play important roles in maintaining membrane fluidity and synthesizing eicosanoids, signaling molecules involved in various physiological processes [141]. The ingestion of LA forms γ-linolenate, eicosatrienoate, and AA [141,147]. From ALA, the human body obtains EPA and DHA [143]. They are precursors to inflammation-fighting eicosanoids [139,148,149]. Because they use the same enzymes, the metabolic pathways converting LA to AA and ALA to EPA are competitive [143,150,151,152]. Because of this competition, the omega-3 to omega-6 PUFA ratios and the relative availability of these substrates directly influence the types of eicosanoids synthesized (Figure 4) [143,150,151,152]. Nowadays, an omega-6 PUFA-rich diet hampers endogenous EPA and DHA synthesis, resulting in extremely inefficient production [143,151,152,153,154]. The limited endogenous synthesis of EPA and DHA due to competitive metabolic pathways necessitates adequate dietary intake. This highlights the importance of both intrinsic metabolic processes and external dietary sources in maintaining optimal sensory and behavioral outcomes.

### 3.3. The Role of PUFAs in Inflammation

PUFAs play a pivotal role in promoting and resolving inflammation [27,137,155]. The process of inflammation is divided into two phases, initiation and resolution [137,156,157,158]. Pathogens, cellular damage, and toxins trigger inflammatory responses in affected tissues [137,158,159]. Inflammation is initiated by eicosanoids, which are potent signaling molecules [160,161]. In response to stimuli, phospholipase A2 releases AA from membrane phospholipids [137,162]. Cyclooxygenase (COX), also called prostaglandin H2 synthase, a bifunctional enzyme of the endoplasmic reticulum, sequentially converts free AA to PGH2, the precursor of various prostaglandins and thromboxanes [163,164]. COX has two catalytic functions, namely COX activity, which uses oxygen to convert arachidonate to PGG2, and peroxidase activity, which further transforms PGG2 into PGH2 [163,164,165]. In general, the two isozymes of PGH synthase are denoted COX-1 and COX-2. In most tissues, COX-1 is constitutively synthesized to maintain gastric tissue health, renal homeostasis, and platelet aggregation, whereas in a minority of tissues, activated immune and inflammatory cell products induce COX-2 production [164,166]. The increased synthesis of series-2 prostaglandins, prostaglandins with two double bonds that are derived from arachidonate, induces COX-2-mediated inflammation, pain, swelling, and vasodilation [167,168]. Inflammation is initiated through a complex pathway, allowing our bodies to fight off bacteria, viruses, and other toxins, as well as remove damaged tissue.

**Figure 5:**
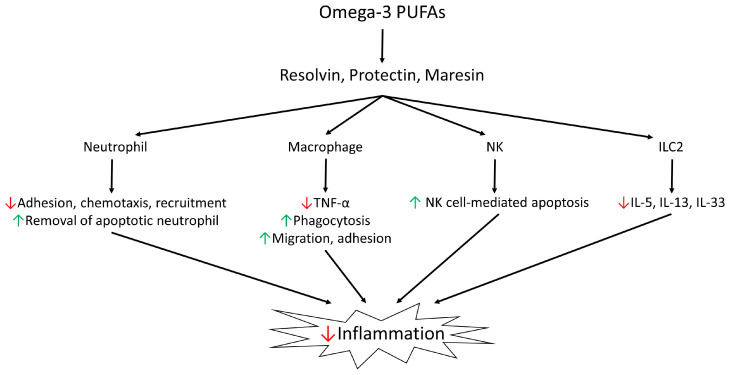
Omega-3 polyunsaturated fatty acids (PUFAs) exert anti-inflammatory effects by regulating a variety of immune cells. Omega-3 PUFAs are essential nutrients that serve as precursors to lipid mediators, including resolvins, protectins, and maresins, collectively known as “specialized pro-resolving mediators (SPMs).” These mediators play a pivotal role in resolving inflammation through multiple mechanisms. In neutrophils, SPMs inhibit adhesion, chemotaxis, and recruitment while enhancing the removal of apoptotic cells, thereby preventing further tissue damage. In macrophages, SPMs decrease the production of the proinflammatory cytokine TNF-α, increase phagocytosis, and promote migration and adhesion, thereby facilitating neutrophil clearance. As for natural killer (NK) cells, SPMs increase the NK cell-mediated apoptosis of eosinophils and neutrophils, a noninflammatory mechanism for cell removal from tissues, consequently promoting the resolution of an inflammatory response. In addition, SPMs regulate innate lymphoid cell type 2 (ILC2) by decreasing the secretion of proinflammatory cytokines, such as IL-5, IL-13, and IL-33. Through several intricate mechanisms, they prevent further recruitment of immune cells to the site of inflammation, effectively curbing the influx of leukocytes that can exacerbate tissue damage. Moreover, SPMs promote the sequestration of proinflammatory cytokines, interrupting the signaling pathways that perpetuate the inflammation response. In addition to these anti-inflammatory actions, SPMs enhance the clearance of apoptotic cells and cellular debris. This process is crucial for preventing secondary necrosis, which can lead to chronic inflammation and further tissue injury. Together, these actions help restore tissue homeostasis during the resolution of inflammation and facilitate the restoration of normal tissue architecture and function.

The inflammatory response should diminish as soon as the source of irritation is controlled, allowing the tissue to return to its normal state. During the resolution phase of inflammation, various mediators are coordinated and regulated. Prominent among these are several anti-inflammatory series-3 prostaglandins and thromboxanes derived from the omega-3 family [169,170]. Several studies have also highlighted the role of SPMs, such as lipoxins (e.g., LXA4), resolvins (e.g., RvD1), protectins, and maresins, in the resolution phase [171,172,173] (Figure 4). These eicosanoids, also derived from EFAs, prevent excessive inflammation and facilitate the removal of debris, microbes, and apoptotic cells, thereby restoring tissue balance and function [173,174]. Unlike traditional anti-inflammatory treatments that broadly suppress natural inflammatory responses, leading to immune suppression and other side effects, SPMs specifically target the resolution phase of inflammation to promote healing without compromising the immune response [171,172,173]. As SPMs are derived from essential PUFAs and exhibit great potential in the treatment of inflammation and the promotion of healing, maintaining an adequate intake of EFAs is crucial. These EFAs, which include omega-3 and omega-6 fatty acids, are fundamental for the production of SPMs. Ensuring sufficient dietary sources of EFAs supports the ability of the body to produce these beneficial mediators, thus enhancing their therapeutic effects (Figure 5). Consequently, incorporating foods rich in these fatty acids, such as fatty fish, flaxseeds, and walnuts, is important for optimizing inflammatory responses and facilitating tissue repair.

## 4. The Role of Endogenous and Dietary PUFAs

### 4.1. The Role of Endogenous PUFAs in Olfactory Function and Related Behaviors

At present, there is a paucity of data analysis regarding the impact of endogenous PUFAs on olfactory function. However, an unbalanced PUFA ratio may cause olfactory disturbances, particularly with lower and higher levels of omega-3 and omega-6 PUFAs, respectively [175,176,177]. This imbalance may potentially explain the associations between PUFAs and olfactory dysfunction caused by inflammatory circumstances. In animal studies investigating the learning ability of olfactory discrimination in omega-3 PUFA-depleted rats, tissue levels of DHA—a long-chain omega-3 fatty acid—were significantly reduced in the olfactory bulb, piriform cortex, and neocortex, leading to prolonged learning times [178]. Other studies further explored the endogenous PUFA concentrations in mice receiving olfactory bulbectomy (OB); it also turned out that the bulbectomized mice had a decreased omega-3 to omega-6 PUFA ratio [176]. Furthermore, endogenous omega-3 PUFAs improved OB-induced depressive behaviors by restoring lipid metabolism and inhibiting the inflammatory response [176]. In human studies, gas and liquid chromatographies were employed to analyze the composition of lipids in the nasal mucosa [177]. According to Khoury et al., nasal AA, an omega-6 PUFA, is positively associated with olfactory deficiency (Table 2) [177]. The above evidence has provided insights into the potential association between PUFAs and smell, as well as the possible etiology and explanation for anosmia.

### 4.2. Dietary Intake of PUFAs and Their Potential Benefits for Olfactory Function

Given that the profile of endogenous PUFAs plays a pivotal role in olfaction and neural transduction, the dietary intake of PUFAs, particularly in the short term, could potentially mitigate the effects of endogenous PUFA deficiencies on olfactory function and theoretically aid in the improvement of smell, to some extent [38]. The previous literature indicates that a lack of dietary omega-3 PUFA intake during the perinatal period can negatively alter the sensitivity of the olfactory mucosa in mice, potentially leading to disturbances in olfactory function [181]. This underscores the crucial role of omega-3 PUFAs in maintaining healthy olfactory and neural functions, highlighting their potential as a therapeutic intervention to support sensory health and mitigate olfactory disturbances, though further research is necessary to confirm its efficacy.

Regarding the treatments of olfactory dysfunction, a common clinical presentation in neurodegenerative diseases such as mild cognitive impairment and Alzheimer’s disease, animal studies have yielded promising results [40]. For example, research involving APOE-transgenic mice demonstrated that olfactory bulb atrophy was significantly alleviated when the mice were fed diets rich in omega-3 PUFAs, particularly DHA [40]. This improvement was likely due to the anti-inflammatory properties of omega-3 PUFAs and their ability to reduce cell death, thereby potentially delaying the progression of olfactory dysfunction [40]. In human research, a 5-year longitudinal study conducted by Gopinath et al., which involved 1331 elderly patients, investigated the effects of fat-rich diets on olfactory dysfunction [179]. The findings indicated that a higher consumption of nuts, fish, and omega-6 PUFAs was associated with reduced odds of olfactory impairment in older adults (Table 2) [179]. In addition, a randomized controlled trial explored the effects of omega-3 PUFAs on olfactory dysfunction in patients undergoing sellar and parasellar tumor resection [180]. This study concluded that omega-3 PUFAs appeared to provide protective benefits against postoperative olfactory disturbances, suggesting that they might be a valuable option for patients during the recovery period (Table 2) [180]. As indicated by the aforementioned research findings, omega-3 PUFAs, owing to their anti-inflammatory properties and ability to reduce cell death, may offer protective and restorative benefits for olfactory dysfunction, both in neurodegenerative diseases and postoperative recovery.

Although there are relatively few studies specifically focusing on the impact of dietary PUFA intake on olfactory function, the available evidence suggests that dietary PUFAs positively influence olfactory function [179,180]. However, since current data on the effects of dietary PUFA intake on olfactory function reveal inconsistent results, further research is required to better elucidate these effects and provide more definitive conclusions.

## 5. PUFAs as Treatments for COVID-19-Induced Anosmia

### 5.1. *The Therapeutic Mechanisms of PUFAs for COVID-Induced Anosmia*

It has been reported that most mammals have similar lipid compositions of olfactory mucosa and olfactory bulbs [175,177,182,183]. The distribution of PUFAs in the olfactory mucosa of rodents has been extensively investigated, and a significant presence of omega-3 and omega-6 PUFAs was observed [182]. Specifically, omega-3 PUFAs constitute 13% of the total lipids, whereas omega-6 PUFAs account for 23% [182]. Similar patterns have been observed in the olfactory bulb of rats, where omega-3 PUFAs comprise 20% and omega-6 PUFAs make up 16% of the total lipid content [175]. Furthermore, PUFAs account for approximately 30% of the total fatty acid content in human nasal mucosa. However, omega-3 and omega-6 PUFAs account for 3% and 26% of the total lipid content in humans, respectively [177]. These suggest that while PUFAs are crucial across species, the specific balance between omega-3 and omega-6 may vary. The presence of PUFAs in olfactory tissues is important, as these fatty acids play a pivotal role in maintaining the fluidity and functionality of cell membranes. Fluidity is essential for the proper functioning of olfactory receptors, which detect and transmit scent information to the brain. In addition, PUFAs participate in anti-inflammatory processes, which protect olfactory tissues from damage and support overall sensory health.

**Figure 6:**
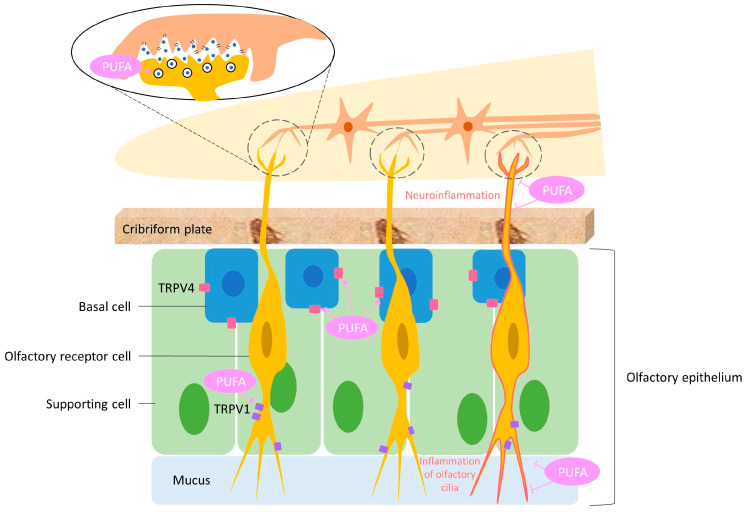
Biomolecular mechanisms of polyunsaturated fatty acid (PUFA) treatment for coronavirus disease 2019 (COVID-19)-induced anosmia. PUFAs are essential for maintaining optimal neurotransmission and facilitating rapid, precise information transfer across the nervous system. They achieve this by regulating the availability of synaptic vesicles at presynaptic terminals. Meanwhile, the dysregulation of the transient receptor potential vanilloid (TRPV) channels may lead to anosmia. Within the olfactory epithelium, PUFAs modulate the function and responsiveness of TRPV1 and TRPV4 channels in olfactory receptor cells and basal cells, respectively. This modulation is crucial, as TRPV channels play a pivotal role in sensory perception and neuronal excitability within the olfactory system. Furthermore, PUFAs exhibit significant anti-inflammatory properties that provide neuroprotective benefits by alleviating neuroinflammation and preventing neural injury, which are vital in combating anosmia, particularly in conditions such as COVID-19. The dual role of PUFAs in regulating inflammation and neuronal excitability in the olfactory epithelium helps maintain both the structural integrity and functional capabilities of the olfactory system. This regulation supports the potential recovery and restoration of olfactory function, highlighting PUFAs as promising treatments for olfactory disorders.

Numerous mechanisms have been identified regarding how PUFAs facilitate olfaction, including neurotransmission, neuroprotection, and anti-inflammation [36,37,38,39,40]. In neurotransmission, previous studies on *Caenorhabditis elegans (C. elegans)* have demonstrated that PUFAs support efficient endocytosis [37], the process that retrieves synaptic vesicle components from the plasma membrane [184]. This ensures an adequate number of releasable synaptic vesicles [185]. PUFAs also facilitate the effective transport of synaptic vesicle precursors from the trans-Golgi apparatus to presynaptic sites [37,186,187]. This helps prevent the accumulation of vesicular components in the cell body and axons [37]. Moreover, PUFAs are crucial for the proper biogenesis and maturation of synaptic vesicle precursors as they move through the endoplasmic reticulum and Golgi apparatus [188]. By maintaining the optimal membrane curvature and aiding in vesicle budding and fusion, PUFAs ensure efficient vesicle biogenesis [37]. Thus, PUFAs help maintain efficient neural transmission by balancing the presynaptic vesicles of neurons [37]. Another probable pathway involves the modulation of TRPV channels, part of the TRP superfamily responsible for neural transduction [64,189]. PUFAs interact with OSM-9, a TRPV channel protein, in the sensory neurons of *C. elegans* [189,190]. In *C. elegans*, OSM-9 plays a pivotal role in sensory transduction and adaptation processes, particularly in response to environmental stimuli, such as odors [189,190]. PUFAs can directly activate the OSM-9 channel, which is important for mediating sensory responses and adapting to prolonged odor exposure [39,191]. Additionally, PUFAs may function downstream of the nuclear translocation of EGL-4, a cGMP-dependent protein kinase, to promote sensory adaptation [191,192,193]. This suggests that PUFAs not only activate OSM-9 but also modulate its activity after key intracellular signaling events [39]. In summary, PUFAs participate in the regulation of both upstream and downstream TRPV signaling, facilitating the discrimination and adaptation of smells [38,39]. Through multiple pathways, PUFAs play a critical role in ensuring efficient neurotransmission (Figure 6).

Meanwhile, the well-documented anti-inflammatory properties of PUFAs, particularly omega-3, may provide significant neuroprotective benefits to both the brain and olfactory system, thereby supporting olfactory function [36,40]. Research indicates that omega-3 PUFAs confer neuroprotection through several mechanisms. Specifically, EPA and DHA have been demonstrated to enhance mitochondrial function [194,195], which is critical for energy production and cellular survival. Moreover, DHA supplementation has been found to boost antioxidant parameters, reduce oxidative markers, and decrease the levels of lipid peroxides and reactive oxygen species, all of which contribute to the maintenance of synaptic integrity and plasticity [135,196]. Furthermore, omega-3 PUFAs modulate inflammatory responses, thereby protecting cells from damage and maintaining normal replacement [197,198]. Collectively, these effects contribute to the maintenance of olfactory function [36]. Thus, omega-3 PUFAs are crucial for neuroprotection and anti-inflammation, which serve as possible mechanisms for the treatment of COVID-19-induced anosmia (Figure 6). The aforementioned mechanisms suggest that PUFAs have the potential to support olfactory function and counteract COVID-19-induced anosmia. Further research into these mechanisms is warranted to deepen our understanding and pave the way for effective therapeutic interventions in clinical settings. We believe that treatments targeting TRPV channels could aid in restoring olfactory function, along with anti-inflammatory agents that reduce inflammation in the olfactory system.

### 5.2. The Potential Therapeutic Role of Omega-3 PUFAs in Post-COVID-19 Olfactory Dysfunction

The rapid spread and evolution of COVID-19 has globally necessitated the development of effective antiviral therapeutics. In particular, anosmia has emerged as one of the public health concerns following the ongoing COVID-19 pandemic [12,199,200]. After a COVID-19 infection, the patient’s immune system may become activated, leading to both short- and long-term olfactory dysfunction [8,72,114,201]. A virus-induced inflammatory response may cause anosmia by damaging the smell neural pathways and the olfactory epithelium [202,203,204,205]. This significantly affects the patients’ quality of life for extended periods of time.

Despite the paucity of research, we suggest that PUFAs help patients with COVID-19-induced anosmia recover their olfactory function. PUFAs play a pivotal role in viral infections, owing to their potential health benefits [206,207,208]. Previous studies have suggested that omega-3 PUFAs regulate inflammation and immune response by influencing the production of lipid mediators and cytokines [209,210]. Thanks to their anti-inflammatory properties, PUFAs may help alleviate respiratory symptoms and reduce COVID-19-associated complications [211,212,213]. The potential therapeutic role of PUFAs in managing COVID-19 symptoms opens up future research avenues focused on the optimization of their use in clinical settings.

Recent studies have suggested that omega-3 PUFA dietary supplementation accelerates recovery, prevents severe complications, reduces hospital stays, shortens intensive care unit stays, and ultimately decreases COVID-19-associated mortality [207,214]. Omega-3 PUFAs provide these benefits through SPMs [171,172], which play a critical role in inflammation resolution by actively terminating the inflammatory response [174,215]. SPMs reduce the intensity of inflammation by mitigating cytokine storms, which are often observed in severe COVID-19 cases [171,216,217,218,219,220]. In addition, they promote tissue repair and help maintain homeostasis [221,222]. Therefore, omega-3 PUFA supplementation shows significant promise in improving the clinical outcomes of patients with COVID-19 by leveraging the anti-inflammatory and healing properties of SPMs.

In a randomized controlled trial conducted by Hernandez et al., 58 patients with post-COVID anosmia exhibited enhanced recovery when they took omega-3 PUFA supplements along with olfactory training [35]. This suggests that the dietary intake of omega-3 PUFAs provides therapeutic benefits for post-viral anosmia and supports olfactory rehabilitation (Table 2) [35]. Similarly, a supervised randomized prospective experiment conducted at the hospital of the Faculty of Medicine, Benha University, showed that a combination of vitamin D and omega-3 fatty acids significantly reduced proinflammatory cytokines and improved olfaction after COVID-19 infection [34]. These results indicate that omega-3 PUFAs may exert synergistic effects with other nutrients to support immune function and attenuate inflammation (Table 2) [34]. However, a prospective, placebo-controlled, randomized clinical trial conducted by Lerner et al., showed negative results [41]. The experimental group received 2 g of omega-3 PUFA supplements daily, while the control group received an identical placebo for 6 weeks [41]. The study did not confirm the efficacy of omega-3 PUFA supplementation for persistent COVID-19-related olfactory dysfunction (Table 2) [41], highlighting the need for further research to explore more effective treatment options.

### 5.3. Potential Reasons for the Lack of Efficacy of PUFAs in Improving Olfactory Function

PUFAs have shown mixed results in improving olfactory function, with seemingly supportive studies containing notable limitations. For example, the study conducted by Hernandez et al. was limited by a relatively low sample size, lack of a placebo control group, a short follow-up period, and baseline differences between the two groups [35]. Additionally, the research by Eldsouky et al. evaluated only a combination of vitamin D and PUFAs, leaving it unclear whether the observed positive effects were due to PUFAs or solely vitamin D [34]. It is important to acknowledge these limitations when interpreting the findings. The study by Lerner et al., which reported negative results, also suggested several reasons why omega-3 PUFA supplementation may have failed to show a significant effect in treating patients with COVID-19-related olfactory dysfunction. Factors such as the short duration of intervention, the chronic nature of the dysfunction, a small sample size, potential confounding factors from other treatments, and measurement limitations may have contributed to this outcome [41].

Currently, the effectiveness of PUFAs in treating COVID-19-induced anosmia remains inconclusive for several reasons. Firstly, anosmia caused by COVID-19 is a complex condition that likely involves multiple mechanisms, such as viral damage to olfactory neurons, inflammation, and disruptions in the central nervous system. PUFA supplementation might not effectively address all these underlying causes, limiting its therapeutic impact. Additionally, individual variability in patient responses, influenced by factors such as genetic predisposition, metabolism, and the severity of anosmia, could lead to inconsistent outcomes. Furthermore, the dosage and duration of PUFA supplementation may have been inadequate to produce a significant therapeutic effect, especially in cases of severe or prolonged anosmia. While PUFAs are known for their anti-inflammatory and neuroprotective properties, their lack of specificity in targeting the olfactory pathways affected by COVID-19 could diminish their effectiveness in restoring olfactory function. Moreover, other treatments, medications, or underlying health conditions could have interfered with the potential benefits of PUFAs, complicating the ability to detect positive effects. Finally, the current evidence base, primarily consisting of preliminary studies with potential design limitations, such as a lack of a placebo control or small sample sizes, may not provide sufficient support for the efficacy of PUFAs in treating COVID-19-induced anosmia.

However, we still believe that PUFA-based therapies hold potential in treating COVID-19-induced anosmia. Future studies should focus on large-scale, placebo-controlled, longitudinal assessments to determine the long-term impact of dietary PUFA intake on post-COVID-19 olfactory dysfunction.

## 6. Conclusions

Anosmia remains a persistent issue for a subset of COVID-19 patients, with some continuing to suffer from olfactory dysfunction long after the acute phase of the infection has passed. We have differentiated between the anti-inflammatory properties of omega-3 PUFAs and the pro-inflammatory effects typically associated with omega-6 PUFAs, emphasizing the complexity of PUFA interactions in the context of inflammation. While omega-3 PUFAs have shown potential in enhancing olfactory function through mechanisms such as improved neurotransmission, neuroprotection, and anti-inflammatory effects, the current body of evidence remains inconclusive. Notably, a placebo-controlled, randomized clinical trial did not demonstrate a significant effect of omega-3 PUFA supplementation on COVID-19-induced anosmia. Moreover, the limitations of the existing studies, such as small sample sizes, a lack of rigorous controls, brief follow-up periods, and the use of combination therapies, hinder the ability to draw definitive conclusions. These limitations underscore the need for future research that addresses these gaps through well-designed, large-scale trials. Such efforts are essential not only for establishing the therapeutic efficacy of omega-3 PUFAs in treating post-COVID-19 anosmia but also for advancing broader strategies in the management of inflammatory and viral infections.

## Figures and Tables

**Table 1:** Classification and major functions of polyunsaturated fatty acids (PUFAs).

Type of PUFA	Chemical Name	Major Sources	Major Functions
Omega-3	α-Linolenic acid (ALA)	Flaxseed, soybean oils	Anti-inflammatory [138,139]
Omega-3	Eicosapentaenoic acid (EPA)	Deep-sea fish, algae	Anti-inflammatory [140]
Omega-3	Docosahexaenoic acid (DHA)	Deep-sea fish, algae	Neuroprotection [140]
Omega-6	Linoleic acid (LA)	Sunflower oils, nuts	Precursor of AA [141]
Omega-6	Arachidonic acid (AA)	Meat, eggs	Precursor of inflammatory mediators [142]

**Table 2:** Human studies on polyunsaturated fatty acids (PUFAs) and olfactory function.

Study	Sample Size	Type of PUFAs	Results
Khoury et al., 2021 [177]	23 patients	Omega-6	High level of nasal arachidonic acid might be associated with hyposmia [177]
Gopinath et al., 2015 [179]	1331 elderly individuals	Omega-3 and omega-6	Higher omega-6 PUFA intake is associated with lower incidence of olfactory dysfunction [179]
Yan et al., 2020 [180]	110 patients with sellar or parasellar tumors undergoing endoscopic resection	2000-mg omega-3 supplementation daily	Omega-3 supplementation appears to be protective for the olfactory system during the healing period [180]
Hernandez et al., 2022 [35]	58 patients	2000-mg omega-3 supplementation daily	Omega-3 supplementation may be a useful adjunct therapy for post-viral olfactory dysfunction [35]
Eldsouky et al., 2023 [34]	226 patients, with 113 individuals in each group (control and treatment)	1.2 g/day omega-3 PUFAs	Omega-3 PUFAs reduce inflammatory markers and improve anosmia from coronavirus disease 2019 [34]
Lerner et al., 2023 [41]	117 patients, with 57 patients in the treatment group and 60 in the placebo group.	2 g/day omega-3 PUFAs	Omega-3 supplementation did not show a statistically significant improvement in patients with COVID-19-related olfactory dysfunction [41]
